# Single-cell sequencing technology applied to epigenetics for the study of tumor heterogeneity

**DOI:** 10.1186/s13148-023-01574-x

**Published:** 2023-10-11

**Authors:** Yuhua Hu, Feng Shen, Xi Yang, Tingting Han, Zhuowen Long, Jiale Wen, Junxing Huang, Jiangfeng Shen, Qing Guo

**Affiliations:** 1 Department of Oncology, The Affiliated Taizhou People’s Hospital of Nanjing Medical University, Taizhou School of Clinical Medicine, Nanjing Medical University, Taizhou, 225300 Jiangsu China; 2 Graduate School, Dalian Medical University, Dalian, 116044 Liaoning China; 3 Department of Neurosurgery, The Affiliated Taizhou People’s Hospital of Nanjing Medical University, Taizhou School of Clinical Medicine, Nanjing Medical University, Taizhou, 225300 Jiangsu China; 4 Department of Radiation Oncology, Cancer Center, West China Hospital, Sichuan University, Chengdu, 610041 China; 5 Department of Thoracic Surgery, The Affiliated Taizhou People’s Hospital of Nanjing Medical University, Taizhou School of Clinical Medicine, Nanjing Medical University, Taizhou, 225300 Jiangsu China; 6 Department of Cardiology, The Affiliated Taizhou People’s Hospital of Nanjing Medical University, Taizhou School of Clinical Medicine, Nanjing Medical University, Taizhou, 225300 Jiangsu China

**Keywords:** Single-cell sequencing, Epigenome, Multi-omics, Tumor heterogeneity

## Abstract

**Background:**

Previous studies have traditionally attributed the initiation of cancer cells to genetic mutations, considering them as the fundamental drivers of carcinogenesis. However, recent research has shed light on the crucial role of epigenomic alterations in various cell types present within the tumor microenvironment, suggesting their potential contribution to tumor formation and progression. Despite these significant findings, the progress in understanding the epigenetic mechanisms regulating tumor heterogeneity has been impeded over the past few years due to the lack of appropriate technical tools and methodologies.

**Results:**

The emergence of single-cell sequencing has enhanced our understanding of the epigenetic mechanisms governing tumor heterogeneity by revealing the distinct epigenetic layers of individual cells (chromatin accessibility, DNA/RNA methylation, histone modifications, nucleosome localization) and the diverse omics (transcriptomics, genomics, multi-omics) at the single-cell level. These technologies provide us with new insights into the molecular basis of intratumoral heterogeneity and help uncover key molecular events and driving mechanisms in tumor development.

**Conclusion:**

This paper provides a comprehensive review of the emerging analytical and experimental approaches of single-cell sequencing in various omics, focusing specifically on epigenomics. These approaches have the potential to capture and integrate multiple dimensions of individual cancer cells, thereby revealing tumor heterogeneity and epigenetic features. Additionally, this paper outlines the future trends of these technologies and their current technical limitations.

## Background

The term “epigenetics” was introduced by Conrad Waddington in 1942 [1] to describe the phenomenon wherein alterations in gene phenotype occur without any corresponding changes in the DNA sequence [2]. At the molecular level, numerous specific modifying proteins are involved in such modifications, including "writers" that catalyze the deposition of specific modifications, "erasers" that catalyze the removal of specific modifications, and "readers" that recognize and bind to modification sites catalyzed by the "writers" and form complexes [3].

The prevailing genetic theory posits that the accumulation of somatic mutations is responsible for the formation of tumors, indicating that cancer cells may emerge from mutations in specific genes that drive oncogenesis [4, 5]. Nevertheless, the lack of mutations with potent oncogenic drivers in various tumor types has prompted researchers to redirect their attention toward non-genetic determinants, such as epigenetic modifications, in the investigation of tumor heterogeneity. Research has demonstrated that changes in epigenetic modifications are significant contributors to malignant biological outcomes, including tumor proliferation, self-renewal, differentiation [6], treatment resistance [7], and tumor metastasis [8].

Despite the advancements brought about by Bulk sequencing in elucidating the mechanisms of epigenetic regulation of tumor heterogeneity, numerous inquiries remain unresolved. Notably, in the investigation of the intricate mechanism of HCC pathogenesis, a handful of studies have highlighted the involvement of ALKBH5 and METTL4 in the pathogenesis of HCC; however, conflicting outcomes have been reported [9–11]. Similarly, in hepatocellular carcinoma, YTHDF2 and METTL3, among other m6A-related proteins, have also yielded conflicting findings [12–16]. The aforementioned phenomenon could potentially be attributed to the intricate cellular heterogeneity of tumors. Regrettably, the comprehensive gene expression data acquired through conventional sequencing techniques may obscure the infrequent cellular subtypes that play a pivotal role in tumor advancement, and the heterogeneity of tumor cells is concealed by the average information of a substantial cell population.

**Fig. 1:**
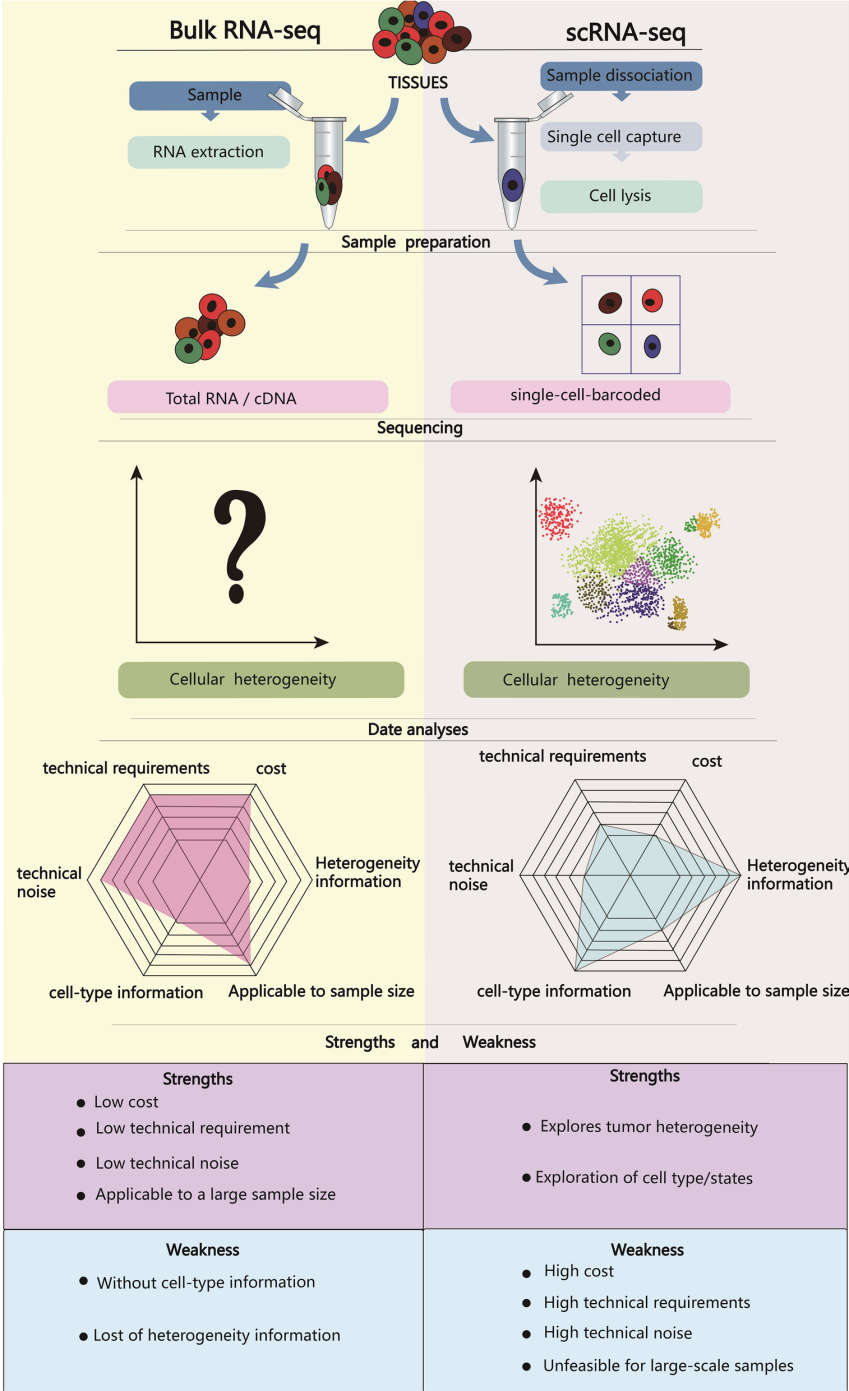
Comparison of the characteristics of single-cell sequencing technology and Bulk sequencing technology

Currently, the field of cancer medicine has transitioned into a phase of precision, whereby the advent of single-cell sequencing technology has provided a promising avenue for investigating the heterogeneity of tumors and the intricate epigenetic regulatory mechanisms that underlie them [17, 18]. In contrast to conventional bulk sequencing, single-cell sequencing offers a notable benefit in its ability to evaluate tumor heterogeneity at the level of individual cells. This overcomes the limitation of traditional sequencing, which can only furnish aggregated data and potentially obscure valid information. Consequently, single-cell sequencing has emerged as a potent instrument for uncovering the epigenetic mechanisms that govern tumor heterogeneity (Fig. 1).

**Fig. 2:**
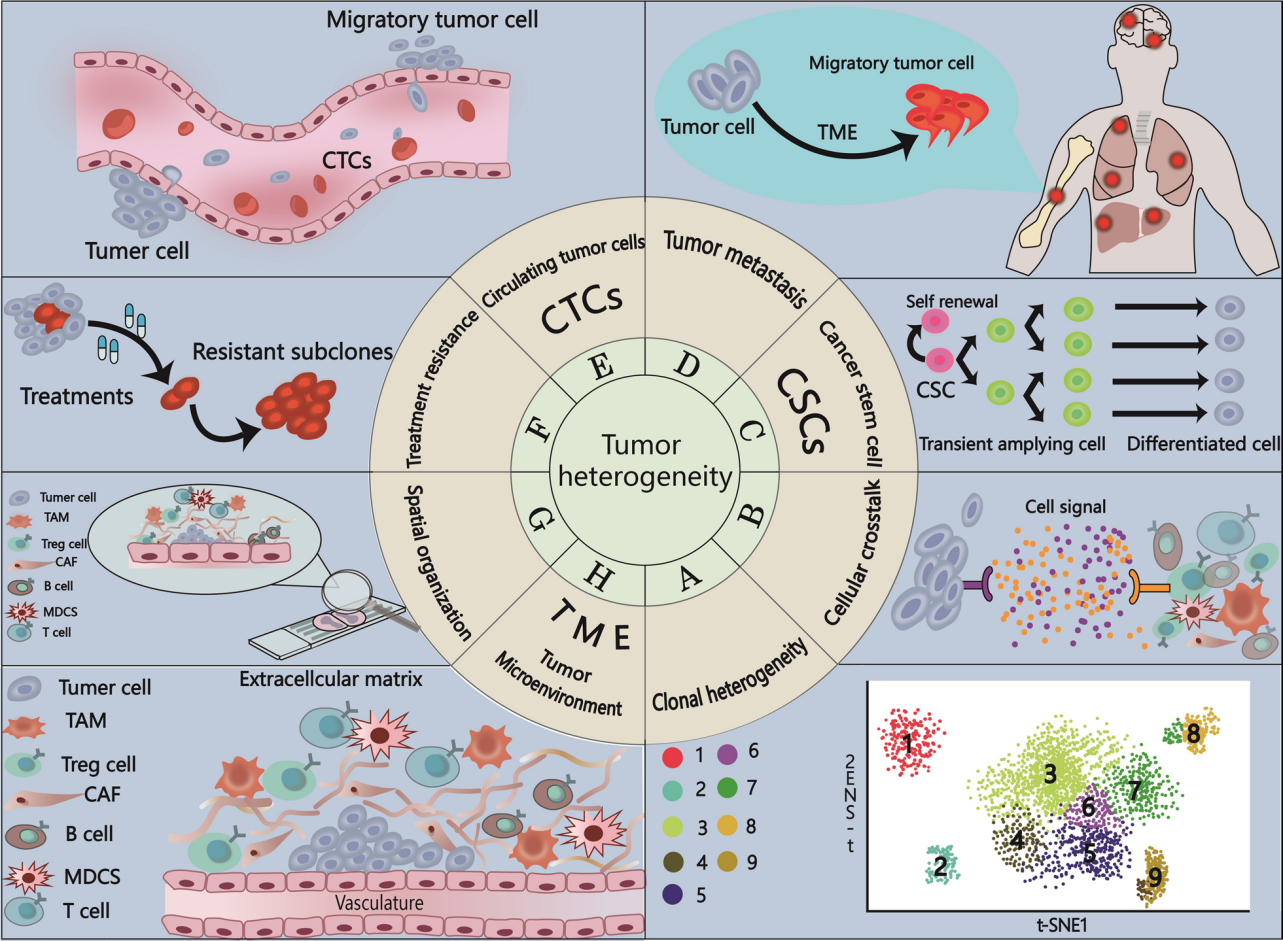
Use of single-cell sequencing allows assessment of epigenetic regulation of tumor heterogeneity not accurately assessed by previous bulk methodologies. Due to the complex cellular heterogeneity of many types of tumors, valid information about individual cells is often masked by the average data of bulk sequencing when using bulk sequencing. However, the emergence of single-cell sequencing has enabled the investigation of epigenetic regulation of tumor heterogeneity, including clonal heterogeneity, cellular crosstalk, tumor stem cells, tumor metastasis, circulating tumor cells, treatment resistance, spatial organization, and tumor microenvironment (TME) mechanisms, which was previously unattainable. **A** Clonal heterogeneity: single-cell sequencing can monitor novel tumor cell subtypes adapted to the tumor microenvironment in the context of epigenetic alterations, revealing the impact of tumor heterogeneity in cancer patients by epigenetic modifications. **B** Single-cell sequencing can infer cellular interactions by correlating the expression of known ligands and receptors, and unravel the epigenomic alterations regulated by this interaction set that lead to tumor development. **C** Cancer stem cells: single-cell sequencing can study the epigenetic background of tumor stem cell differentiation trajectory to predict tumor progression and reveal the heterogeneity of tumor cells. **D** Tumor metastasis: single-cell sequencing can monitor rare cellular mutations that acquire invasiveness, metastasis, immune escape and EMT during tumor development and investigate the mechanisms underlying the epigenetic regulation of this process. **E** Circulating tumor cells: single-cell sequencing can be used to investigate the epigenetic regulation of metastasis by comparing the expression differences between CTCs in blood with metastatic dissemination of tumors and the primary tumor. **F** Treatment resistance: single-cell sequencing allows monitoring rare cellular mutations that acquire treatment resistance in tumors (e.g., intrinsic drug resistance and acquired drug resistance) and investigating the mechanisms of epigenetic regulation of this process. **G** Spatial organization: single-cell sequencing can obtain temporal and spatial information on gene expression, and in situ measurement of the epigenome can better reveal the association between cellular spatial distribution and gene regulation in tumor tissues. **H** Tumor microenvironment (TME): single-cell sequencing can investigate the phenotypic and functional heterogeneity of various cell types caused by epigenomic alterations in the tumor microenvironment (TME) during tumorigenesis and progression

Tumors are intricate and heterogeneous biological systems, and it has been recognized that epigenetics plays a crucial role in cancer initiation and progression. Bulk sequencing alone is insufficient to fully elucidate the intricate epigenetic regulatory mechanisms underlying tumors. Single-cell epigenetic analysis has emerged as a valuable tool for exploring previously uncharted aspects of epigenetic heterogeneity associated with tumor biology, including clonal heterogeneity, the tumor microenvironment, cancer stem cells, circulating tumor cells, treatment resistance, intercellular communication, and tumor metastasis (Fig. 2).

However, there are extensive interactions between epigenomes and other omics, and examining epigenomics exclusively at the single-cell resolution can limit a comprehensive comprehension of epigenetic heterogeneity in tumors. By integrating single-cell epigenomic, transcriptomic, genomic analyses, we anticipate expanding the current understanding of how epigenetic domains influence tumor heterogeneity through a multi-dimensional single-cell epigenetic multi-omics approach. This approach is often unachievable with bulk or single-cell epigenetic sequencing alone.

## Single-cell sequencing technology applied to omics to explore the impact of epigenetic modifications on tumor heterogeneity

Since the first appearance of single-cell transcriptomics technology in 2009 [19], a plethora of such techniques have emerged. With continuous improvements in these technologies, we are now able to perform individualized analyses of specific cellular features, moving from the multi-cellular level of tissues to the single-cell realm, thereby obtaining a more detailed understanding of tumor cell heterogeneity.

In recent years, the advent of numerous bulk and single-cell level epigenetic sequencing methods has provided powerful tools for mapping various epigenetic modifications, such as chromatin accessibility, histone modifications, DNA methylation, and single-cell nuclear organization [20]. These tools have proven instrumental in unraveling the epigenetic mechanisms underlying malignant tumor cells [21].

**Fig. 3:**
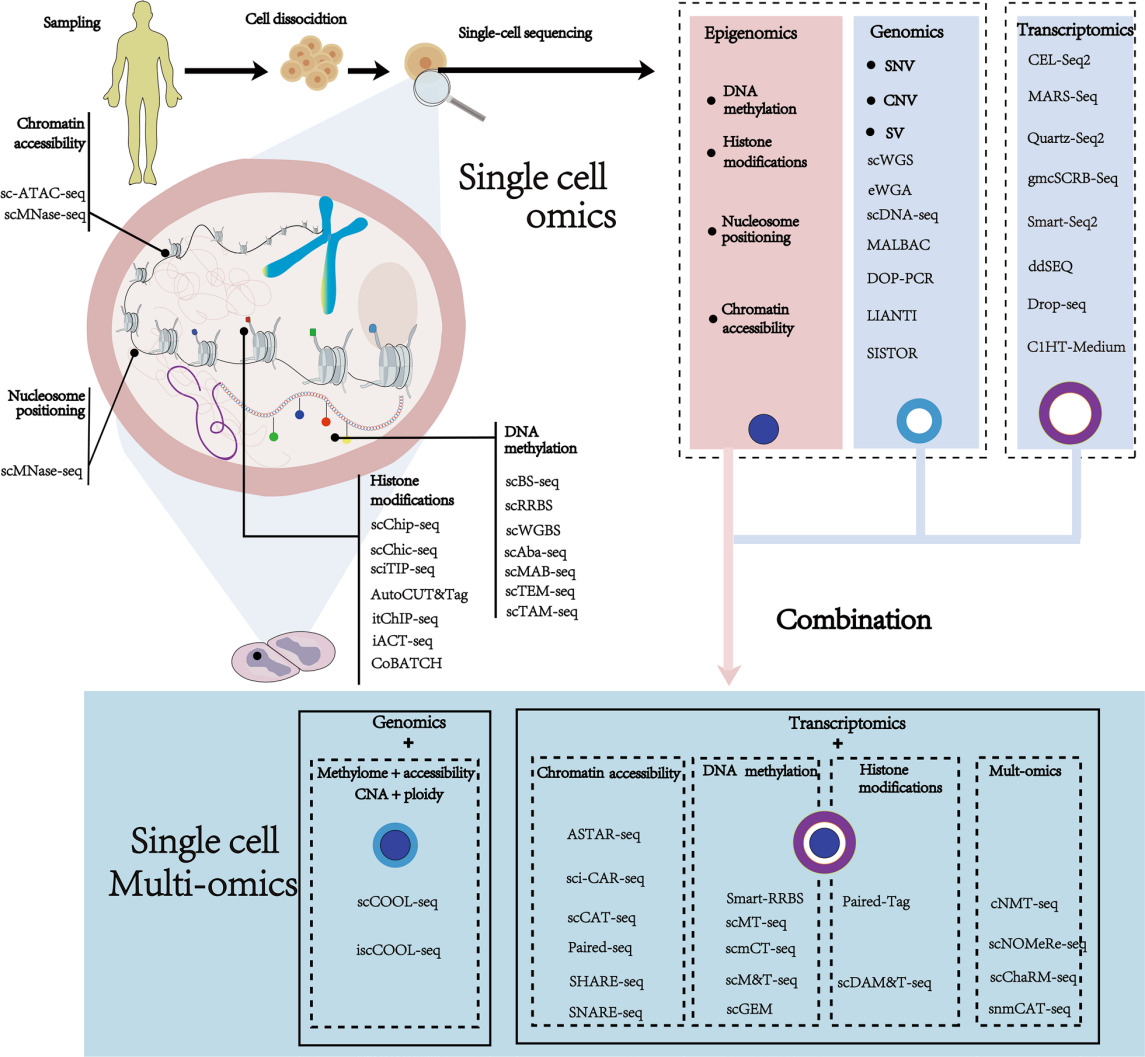
Single-cell mono-omics and single-cell multi-omics sequencing approaches to research the epigenetic mechanisms of cancer

However, studying only the epigenome of tumors is insufficient to fully reveal the complexity of tumor heterogeneity [22]. To address this issue, researchers have proposed integrating epigenomic data with transcriptomic, and genomic data from other omics levels at the single-cell resolution. This comprehensive analysis enables a holistic understanding of the relationship between epigenetic regulation and cancer heterogeneity [23] (Fig. 3).

## Transcriptomics

Transcriptome sequencing is a method used to understand the gene expression profile by sequencing all the expressed genes in a cell. Traditional transcriptome sequencing methods often require a large amount of cell samples, which obscures subtle differences between individual cells within a cell population, especially in highly heterogeneous tumor cells.

Single-cell transcriptome sequencing technology is a high-resolution approach that enables sequencing of mRNA in individual cells. Common techniques for single-cell transcriptome sequencing include single-cell RNA sequencing (scRNA-seq) [24] and single-cell nuclear sequencing (scNuc-seq). These techniques utilize microfluidic chips, microarrays, droplet-based or partition-based approaches to isolate and capture individual cells, perform cDNA synthesis, DNA amplification, and high-throughput sequencing, to obtain transcriptomic information from individual cells and deeply study the heterogeneity and characteristics of tumor cells [25].

Single-cell transcriptome sequencing has broad applications in tumor research. Through this technology, we can identify and define different subgroups and cell types within tumor cells, gaining insights into the cellular heterogeneity within tumors [26]. Additionally, the utilization of scRNA-seq assays to track temporal genealogy at various stages of tumor development can facilitate the identification of key gene mutations that initiate tumor progression, while also providing insights into the interplay between heterogeneity and temporal dynamics [27]. In the study of tumor immune microenvironment, this technology helps to identify and characterize tumor-infiltrating immune cells and immune suppressive cells, predict patient responses to immunotherapy, and provide foundations for the development of immunotherapy strategies [28]. Moreover, single-cell transcriptome sequencing can also predict patient responses to specific treatments, enabling personalized treatment selection, and provide insights into the mechanisms of treatment resistance for theoretical research [29]. Finally, this technology contributes to the discovery and validation of novel tumor-related genes and signaling pathways, providing potential targets and theoretical basis for targeted therapy and new drug development [17, 30, 31]. In summary, single-cell transcriptome sequencing provides a powerful and comprehensive tool for tumor research, greatly advancing our understanding of tumor development and treatment, and providing important scientific foundations for personalized treatment strategies and new drug development.

A study utilized single-cell RNA sequencing to analyze individual cells from pancreatic ductal adenocarcinoma (PDAC) tumors and control tissues, revealing the cellular heterogeneity and progression mechanisms within PDAC. The study identified a high degree of heterogeneity in PDAC, encompassing various malignant and stromal cell types. The malignant subtypes were found to consist of multiple subpopulations with distinct proliferation and migration capabilities. Additionally, the study observed a correlation between a subset of ductal cells and the inactivated state of tumor-infiltrating T cells. These findings provide valuable resources and insights for understanding the intra-tumoral heterogeneity in PDAC and identifying potential biomarkers for anti-cancer therapies [26].

There have also been studies through single-cell RNA sequencing (scRNA-seq) to analyze metastatic lung cancer before and during targeted therapy. They found a diverse and dynamic tumor ecosystem consisting of cancer cells and the tumor microenvironment (TME). The scRNA-seq analysis identified targetable oncogenes in cancer cells and revealed different gene expression patterns in residual disease (RD) and progressive disease (PD) under therapy. RD cells showed a transition to a primitive cell state, while PD cells exhibited upregulated pathways related to kynurenine, plasminogen, and gap junctions. This study also observed the presence of active T-lymphocytes and reduced macrophages in RD, while immunosuppressive cell states characterized PD. These biological features identified through scRNA-seq analysis served as biomarkers for clinical outcomes in independent patient cohorts. This research highlights the impact of therapy-induced adaptations in the multi-cellular ecosystem of metastatic lung cancer on treatment response and patient outcomes [32].

Another study analyzed cancer-associated fibroblasts (CAFs) in gastric cancer using single-cell RNA sequencing. The researchers discovered the heterogeneity of CAFs and their dynamic communication with components of the tumor microenvironment. Four distinct subsets of CAFs were identified, and two subsets, namely inflammatory CAFs (iCAFs) and extracellular matrix CAFs (eCAFs), exhibited enhanced pro-invasive activities and interactions with immune cell subsets. eCAFs were also associated with poorer overall survival in patients. Therefore, inhibiting the activation of these CAF subsets holds promise as a therapeutic strategy for improving the treatment of gastric cancer [33].

After the first single-cell transcriptome sequencing paper was published in 2009 [19], there have been significant improvements in the capacity and resolution of single-cell sequencing libraries. These advancements have enabled us to simultaneously explore whole transcriptome profiles of gene expression in thousands of cells (Table 1).

**Table 1:** Current methods available for single-cell RNA sequencing

Technique	Technical features	Designed by
CEL-Seq	Lower throughput (hundreds to thousands of single cells); linear amplification sequencing method (lower cost); only be used for 3′ end sequencing; introduces barcode sequences; application to cellular heterogeneity and molecular mechanisms; suitable for exploring cellular heterogeneity and molecular mechanisms	[34]
CEL-Seq2	As an upgraded version of CEL-Seq1;introduces UMI (Unique Molecular Identifier) to eliminate sequencing bias introduced by PCR amplification	[35]
MARS-Seq	High throughput (large numbers of single-cell samples); unique molecular tags enable hybrid sequencing of transcriptomes from multiple cells (lower cost); suitable for exploring heterogeneity in tumors and capturing spatial transcriptomic information	[36]
MARS-Seq2	As an upgraded version of MARS-Seq1;introduces UMI (Unique Molecular Identifier) to eliminate sequencing bias introduced by PCR amplification	[37]
Quartz-Seq	High throughput (hundreds to thousands of individual cells); relatively high loss of cells during sample preparation; requires microfluidic chips; high cost; suitable for studying gene expression patterns and cellular heterogeneity in single cells	[38]
Quartz-Seq2	As an upgraded version of Quartz-Seq; highly sensitive and high throughput; technical noise and bias: amplification bias and loss of a portion of low abundance RNA; requires a certain number of cells to obtain sufficient RNA quality	[39]
mcSCRB-seq	The mcSCRB-seq's "multi-channel" allows sequencing of multiple samples (reducing cost per sample), increasing throughput and efficiency; unique barcodes are incorporated during reverse transcription, allowing for the pooling and simultaneous sequencing of multiple cells	[40]
Smart-Seq	Medium throughput (tens to hundreds of individual cells); high initial RNA volume requirements; bias and noise may be introduced during amplification; high cost per sample; captures full-length transcriptome information for detailed analysis of cell types or states with complex transcriptomic regulatory networks	[41]
Smart-Seq2	As an upgraded version of Smart-Seq; Introduction of UMI (Unique Molecular Identifier) sequences and sample-specific index sequences; Smart-Seq2 uses T7 RNA polymerase for amplification with higher amplification efficiency (VS Smart-Seq1 Linear amplification technique)	[42]
Smart-seq3	As an upgraded version of Smart-seq2, Smart-seq3 has 5′ UMI and achieves more efficient sequencing; Smart-seq3 is able to detect more genes, especially low abundance genes; Efficiently removes most of the ribosomal RNA (rRNA)	[43]
ICELL8	High throughput (thousands of cells on a single chip); unique microfluidic chip required; Multi-Hole Options; Sufficient number of cells is required to ensure good capture efficiency	[44]
Drop-seq	Drop-seq is a microdroplet-based technology; high throughput (thousands of single cells can be processed and millions sequenced); Lower sample cost	[45]
inDrop	Drop-seq is a microdroplet-based technology; high throughput (thousands of single cells can be processed and millions sequenced); Lower sample cost; inDrop introduces an indexing technique (compared to Drop-seq) that enables simultaneous sequencing of multiple samples through the introduction of barcoded beads; suitable for exploring cellular heterogeneity	[46]

Single-cell transcriptomics has also been integrated with various novel technologies to provide a more comprehensive understanding of tumor heterogeneity. CITE-seq is an example of such technology that combines transcriptome analysis with phenotypic characterization, allowing simultaneous sequencing of the single-cell transcriptome and indexing of cell surface proteins [47]. CROP-seq, by integrating CRISPR screening with single-cell transcriptome resolution, facilitates comprehensive analyses of complex regulatory mechanisms and different cell populations, enabling high-throughput functional profiling [48, 49]. The LINNAEUS technique enables simultaneous lineage tracing and transcriptomic analysis of numerous single cells, providing a systematic approach for tracing the origins of novel cell types or identifying known cell types under different conditions [50]. These advantages make single-cell transcriptome sequencing technology particularly suitable for studying the heterogeneity of tumor cells.

Table 1 presents the unique advantages and limitations of single-cell transcriptomics in terms of characteristics, throughput, and applicability.

## Epigenomes

The significance of epigenetic regulation in the progression of development and disease is widely recognized. The introduction of single-cell sequencing technologies has facilitated the development of various methods for analyzing epigenetic regulation at different levels, including chromatin accessibility, DNA methylation, histone modifications, and nucleosome positioning, among others. These technologies have enabled researchers to investigate various aspects of epigenetics at the single-cell level [20, 51]. Such techniques have emerged as powerful tools for uncovering the distinctive epigenomic characteristics of rare cellular subtypes and the epigenetic diversity present within cellular populations.

### Chromosome accessibility

Nucleosomes serve as the fundamental structural units of eukaryotic chromatin which consist of DNA wrapped around a core of histone proteins. The chromatin structure is further organized into a three-dimensional arrangement, which includes densely packed regions and more open, accessible regions. These distinct regions of chromatin structure play a crucial role in regulating gene expression. During replication and transcription processes, the tightly packed chromatin structure needs to be opened up to expose specific DNA sequences for regulatory factors to bind and carry out their functions. This opening up of the chromatin structure to allow regulatory factor binding is referred to as chromosome accessibility [52]. It involves the dynamic modulation of the chromatin structure, allowing access to the DNA. Studies have demonstrated that the accessibility of DNA sequences within the chromatin structure can influence gene transcription activity. Changes in chromatin accessibility can either promote or repress gene expression [52, 53]. This process is mediated by various mechanisms, including histone modifications, chromatin remodeling complexes, and interactions with transcription factors.

**Fig. 4:**
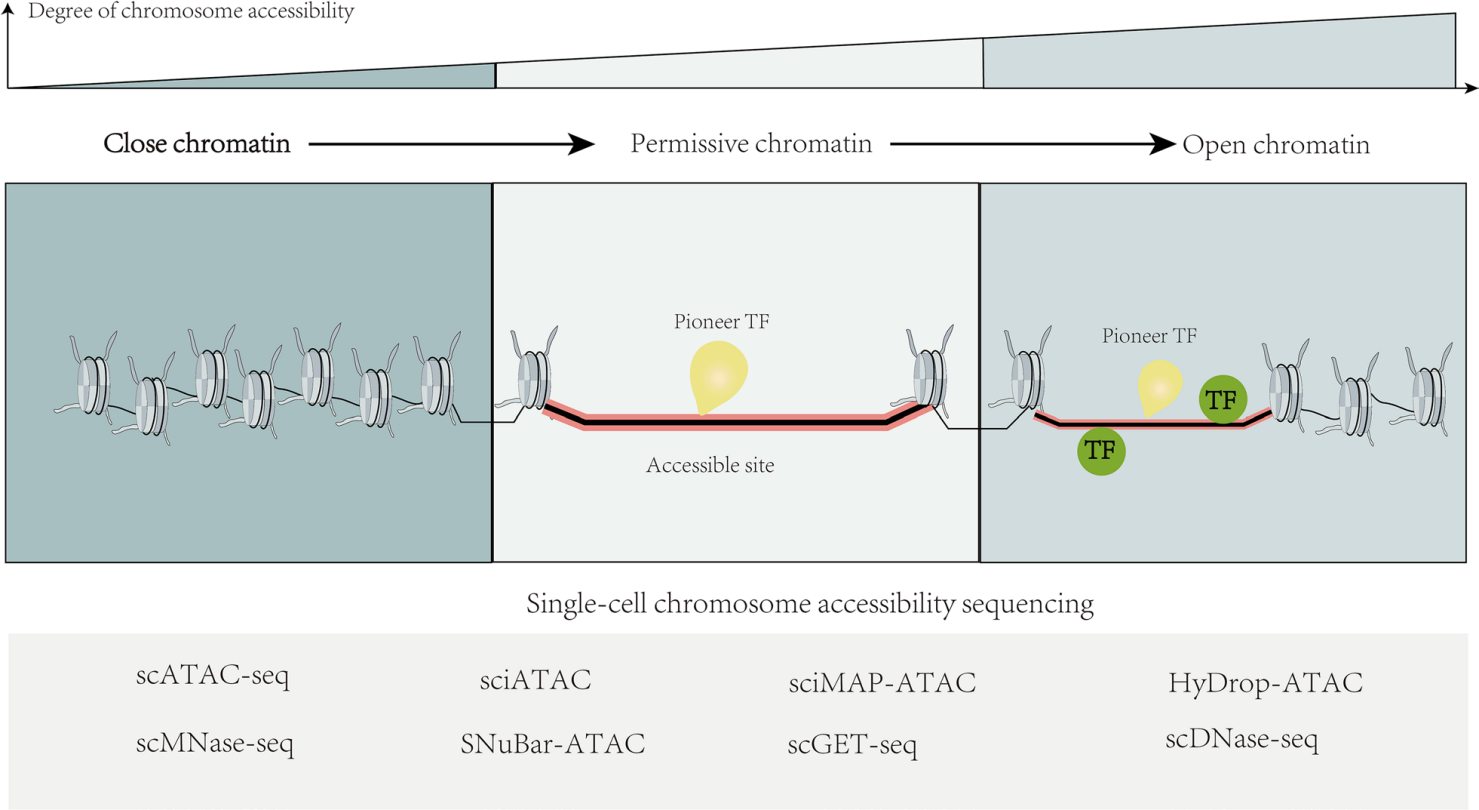
Insights into the dynamic regulation of chromatin accessibility and methods for single-cell chromatin accessibility sequencing

Chromatin accessibility is a field of active research and understanding its impact on gene expression has important implications in various cellular processes and diseases. By studying the accessibility of DNA within chromatin, scientists can gain insights into the mechanisms underlying gene regulation and potentially discover new targets for therapeutic interventions. The field of single-cell chromatin accessibility sequencing has made significant progress, enabling the detailed exploration of chromatin accessibility in individual cells shown in Fig. 4. Among the techniques developed for this purpose, single-cell ATAC-seq (scATAC-seq) has emerged as a powerful method. scATAC-seq leverages the Tn5 transposase’s sensitivity to identify open and accessible chromatin regions [54]. It allows for the investigation of chromatin accessibility landscapes at a single-cell resolution, providing insights into the epigenetic regulation and heterogeneity within tumors.

In addition to scATAC-seq, other techniques have been employed to assess chromatin accessibility at the single-cell level. For example, single-cell DNase-seq (scDNase-seq) utilizes DNase I digestion to identify accessible DNA regions in individual cells [55]. This method enables the detection of regions of open chromatin and provides valuable information about transcription factor binding and regulatory elements. Other new technologies are shown in Table 2.

**Table 2:** Current methods available for chromosome accessibility sequencing

Technique	Technical features	Designed by
scATAC-seq	scATAC-seq enables sequencing of chromatin accessibility of each individual cell; technical complexity; data complicated and high costs	[54]
scMNase-seq	scMNase-seq sequences and analyzes chromatin structure and accessibility in individual cells by enzymatic cleavage of chromatin by micronucleases (MNases); limitations of technical complexity and fragmentation; high cost	[56]
sciATAC	sciATAC is a DNA transposase-based single-cell sequencing technology for analyzing chromatin accessibility in single cells; introduced the strategy of combinatorial indexing; limitations of technical complexity and fragmentation; high cost	[57]
SNuBar-ATAC	SNuBar-ATAC is a technology for measuring single-cell chromatin accessibility and gene expression with high resolution, integrated multiple measurements and microfluidics; data complicated and high costs	[58]
sciMAP-ATAC	SNuBar-ATAC is a technology for measuring single-cell chromatin accessibility and gene expression with high resolution, integrated multiple measurements and microfluidics; limitations of technical complexity and fragmentation; high cost	[59]
scGET-seq	scGET-seq is a hybridized transposase-based sequencing of single-cell genomes and epigenomic transposases, enabling comprehensive probing of open and closed chromatin and simultaneous documentation of underlying genomic sequences	[60]
HyDrop-ATAC	HyDrop-ATAC is a technology for measuring single-cell chromatin accessibility and gene expression with high resolution, direct sequencing and capture of dynamic information (introducing special fluorescent markers); limitations of technical complexity and fragmentation; high cost	[61]

These single-cell chromatin accessibility methods have been widely applied in tumor research, offering insights into the heterogeneity and regulatory dynamics within tumor cell populations. For instance, a study utilized single-cell ATAC-seq technology to analyze the cellular composition and state changes that occur during the transformation from healthy colon to precancerous adenomas to colorectal cancer (CRC). It revealed the cellular composition and state changes that occur during the transformation from healthy colon to adenomas and subsequently to CRC. In the cancerous state, the study observed T cell exhaustion, RUNX1-regulated cancer-associated fibroblasts, and increased accessibility associated with HNF4A motifs in epithelial cells. Furthermore, in sporadic CRC, the DNA methylation changes were strongly anti-correlated with the accessibility changes along this continuum, thus providing additional regulatory markers for molecular staging of polyps [55].

The process of tumor metastasis is a prominent contributor to mortality among individuals diagnosed with cancer [62, 63], and the utilization of scATAC-seq has significantly enhanced our comprehension of the underlying mechanisms involved in tumor metastasis. A study identified novel cell subpopulations with abnormally high CXCL14 expression levels in patients with breast cancer PL by transposase accessible chromatin (ATAC) sequencing (scATAC-seq) of breast cancer negative (NL) and positive lymph nodes (PL), and also identified potential regulators that may be associated with breast cancer lymph node metastasis, improving our understanding of the mechanism of lymph node metastasis of lymph node metastasis and provide a new prognostic marker for breast cancer lymphatic metastasis [64]. Another study confirmed that TCF7 promotes epithelial-mesenchymal transition (EMT) and that activation of EMT is a key process in cancer metastasis by performing single-cell RNA sequencing and scATAC-seq on tumors from patients with low risk of recurrence, high risk of recurrence, and recurrent bladder cancer [65].

The tumor microenvironment encompasses diverse cellular and structural constituents that exert a pivotal influence on tumor advancement and resistance to therapeutic interventions. Moreover, the examination of single-cell chromatin accessibility can be extended to the analysis of the tumor microenvironment. In one study, by performing scRNA-seq and scATAC-seq on ccRCC primary tumor tissues, investigators identified key regulatory molecules in the tumor microenvironment that mediate tumor progression and manipulate immune cell function, and further experimentally validated their role in tumor growth [66]. Another study identified regulatory mechanisms associated with CD8 T-cell depletion by analyzing scATAC-seq profiles of serial tumor biopsies before and after programmed cell death protein 1 blockade [67].Twenty-two human IDH mutant gliomas were analyzed by scATAC-seq, which explained how different subtypes of IDH mutant gliomas maintain different phenotypes and tumor microenvironments despite being derived from a common spectral hierarchy, and found that ATRX regulates glial identity and tumor microenvironment in IDH mutant gliomas [68].

scATAC-seq proves to be a valuable tool for investigating alterations in cellular response to therapeutic interventions throughout the course of tumor treatment. To improve the clinical outcome of CAR-T cell therapy, a study by scATAC-seq of sorted T-cell subsets from seven patient, it was found that IRF7-regulated chronic IFN signaling was associated with poor persistence of CAR-T cells in T-cell subsets and that TCF7 regulators were relevant not only in maintaining naive and early memory T-cell states, but also in maintaining a good phenotype in effector cell lineages play a role [69]. Another study, by scATAC-seq of 12 breast cancer patients, found that the transcription factor GRHL2 cooperates with FOXA1 to initiate endocrine resistance and that epigenetic heterogeneity may contribute to endocrine resistance in breast cancer patients [70].

Table 2 presents the unique advantages and limitations of single-cell chromatin accessibility in terms of characteristics, throughput, and applicability.

### DNA methylation

**Fig. 5:**
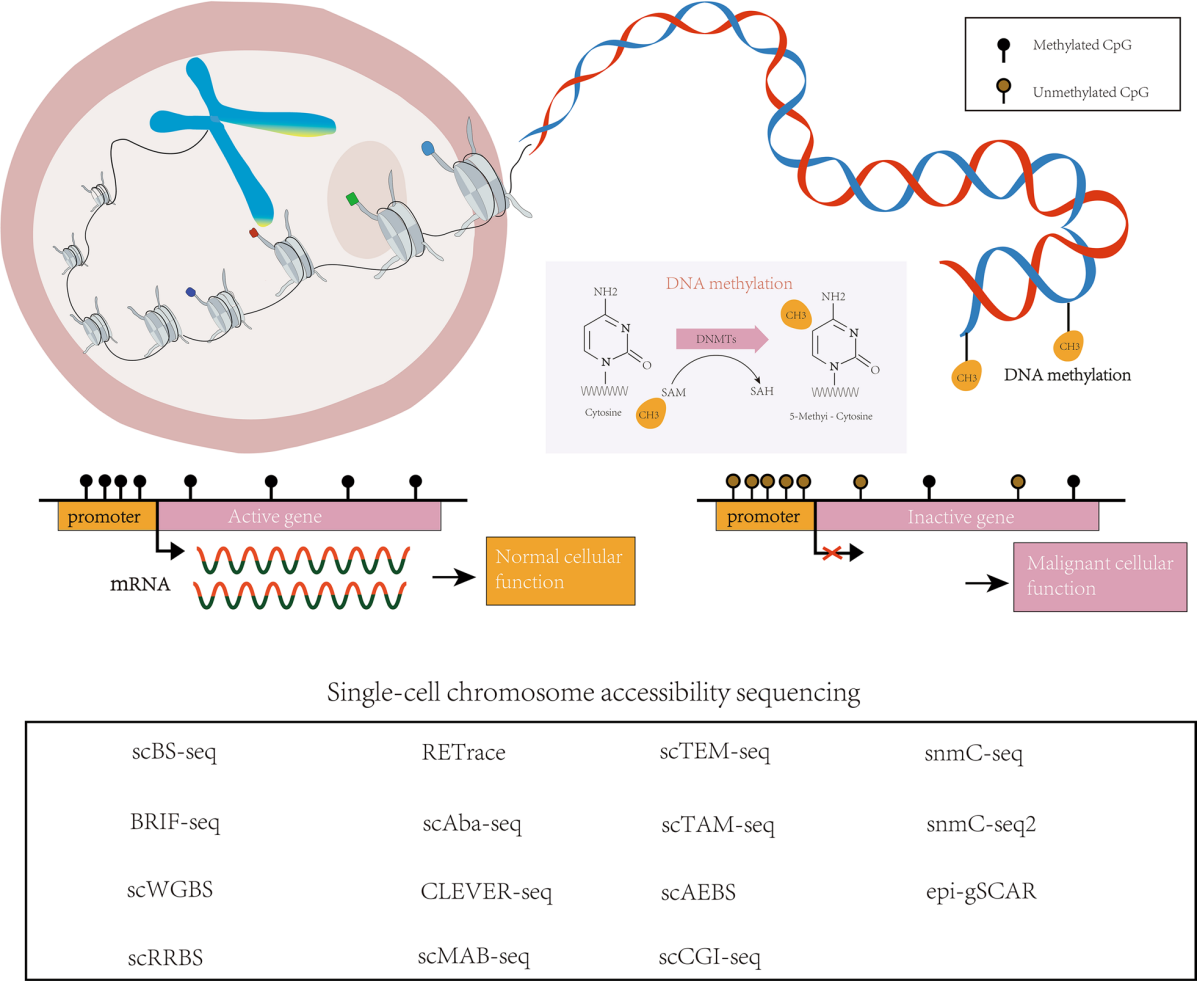
Insights into the dynamic regulation of DNA methylation and methods for single-cell DNA methylation sequencing

DNA methylation is a modification in which –CH3 methylation modification occurs on cytosine bases [71], specifically 5mC (5-methylcytosine) in this article. This methylation process is catalyzed by the DNA methyltransferase (DNMT) family, which adds methyl to the cytosine of the 5′ terminal CpG dinucleotide of human genes. A CpG island is a region in the 5′ → 3′ direction of a DNA sequence that is rich in CG dinucleotides. CpG islands can be found in the promoters of more than two-thirds of all genes and in most cases these CpG islands are in an unmethylated state. CpG islands act as definitive markers of DNA methylation and act as key switches in epigenetic regulation [72], restricting gene expression in the presence of methylation. Studies have shown that methylation of CpG islands plays an important role in transcriptional regulation and is usually altered during malignant transformation. Approximately 5–10% of normal unmethylated CpG island promoters are aberrantly methylated in various cancer genomes. Methylation of promoter CpG islands mediates the phenomenon of gene silencing observed during tumorigenesis [73, 74]. In addition, dysregulated DNA methylation is considered a hallmark of cancer. Genomic demethylation and gene-specific hypermethylation are prevalent in oncogenes and tumor suppressor genes [75–77] (Fig. 5).

With the advancement of next-generation sequencing (NGS) technologies, high-throughput methylation sequencing methods have made significant progress, enhancing the accessibility and efficiency of sequencing. Among these methods, bisulfite sequencing is considered the “gold standard” for DNA methylation analysis due to its high accuracy and single-base resolution [78, 79]. This technology includes whole-genome bisulfite sequencing (WGBS), which evaluates the extent of methylation within CpG islands by converting unmethylated cytosine © to uracil (U) while leaving 5-methylcytosine (5mC) unchanged [80]. WGBS provides in-depth understanding of the DNA methylation patterns across the entire genome and has revolutionized our understanding of DNA methylation. To reduce costs and increase sample throughput, researchers have developed methods that target specific regions for methylation sequencing, such as reduced representation bisulfite sequencing (RRBS) [81]. RRBS utilizes restriction enzymes to enzymatically cleave the genome, reducing its complexity during sequencing and enriching the analysis for important regulatory regions like promoters and CpG islands where detailed methylation analysis can be performed.

However, previous methods relied on bulk sequencing, which averaged the methylation information of cell populations, unable to resolve the heterogeneity present within individual cells. With the development of single-cell sequencing technologies, single-cell methylation sequencing has become feasible. The first method based on single-cell bisulfite sequencing was single-cell methylation genome sequencing (scRRBS), which employs enzymatic cleavage to generate CpG-rich DNA fragments for subsequent library construction and sequencing [82]. However, the harsh conditions used in bisulfite conversion can lead to DNA degradation, resulting in DNA loss and reduced sequencing quality, ultimately affecting the data yield. To address this issue, researchers have developed PBAT (post-bisulfite adaptor tagging) to mitigate the loss caused by degradation. Other single-cell methylation sequencing methods have also been developed, as shown in Table 3.

**Table 3:** Current methods available for single-cell DNA methylation sequencing

Technique	Technical characteristics	Designed by
scBS-seq	scBS-seq is a technique for single-cell DNA methylation analysis with the advantages of single-cell resolution, high-precision detection and integrate multi-omics information; large and complex data; incomplete methylation reactions may lead to low coverage and bias	[83]
BRIF-seq	BRIF-seq is a sequencing method with high read rate and genomic coverage by single strand ligation, MDA amplification and Tn5-based library building of small fragments generated by random amplification	[84]
scWGBS	scWGBS is based on sulfite sequencing technology for single-cell whole-genome DNA methylation analysis with the advantages of single-cell resolution, whole-genome coverage and high resolution	[85]
scRRBS	scRRBS narrows down the analysis of whole-genome DNA methylation sequencing to key CpG sites to achieve downscaling, high-precision detection, and sequencing cost savings; higher coverage in CpG islands and CpG-enriched regions, leading to potential bias	[82]
RETrace	RETrace is a sequencing method that combines microsatellite capture with scRRBS; allowing simultaneous retrospective gene tracing and methylation analysis of single cells	[86]
scAba-seq	scAba-seq is a technique for combinatorial analysis of single-cell antibodies with single-cell resolution, multi-parameter analysis, and high sensitivity; coverage of antibody probes, does not comprehensively reflect the expression of all antibodies; technological complexity	[87]
CLEVER-seq	CLEVER-seq is a sequencing technology for the detection of long-range DNA sequence variation, featuring long-range sequencing, high sensitivity and high resolution; reliance on precise primer design; the need for high efficiency in DNA ligation reactions; and the detection of genomic structural variation and copy number variation	[88]
scMAB-seq	scMAB-seq is a technique for single-cell multi-antibody combinatorial analysis of multiple antibody combinations in a single cell; characterized by multi-antibody analysis, high throughput, and single-cell resolution; limited by antibody probe coverage; technical complexity	[89]
scTEM-seq	scTEM-seq combines transmission electron microscopy technology and sequencing technology for sequencing and analyzing individual cellular ultrastructures with high resolution and structural and transcriptome linkage; relatively low throughput; technical complexity; limited sample processing capacity; high cost	[90]
scTAM-seq	scTAM-seq is a targeted bisulfite-free method that enables targeted high-confidence analysis of DNA methylation in single cells	[91]
scAEBS	scAEBS is based on agarose-embedded bisulfite treatment to investigate DNA methylation at multiple loci by multiplex PCR (multiplex scAEBS)	[92]
scCGI-seq	scCGI-seq is a technique for single-cell CpG island methylation status analysis with single-cell resolution, high resolution, and multi-omics correlation; technical complexity; and coverage is limited by the coverage of the selected CpG island probe	[93]
snmC-seq	snmC-seq is a sequencing technology for DNA methylation profiling of individual cell nuclei with single-cell resolution and DNA methylation resolution; loss of cellular subcellular structural information; low signal-to-noise ratio; high cost per sample	[94]
snmC-seq2	snmC-seq2 is a technique for determining methylation in individual neuronal cells; resolves DNA methylation status at the single-cell level; genome-wide assessment of DNA methylation in single cells; technical complexity; high cost	[95]
epi-gSCAR	epi-gSCAR is a single-tube, bisulfite-free method that allows simultaneous genome-wide analysis of DNA methylation and genetic variation in single cells	[96]

Methylation events have a major impact on the regulation of cell fate, and single-cell DNA methylation sequencing has provided key new insights into the important issue of tumor heterogeneity. For instance, a study utilized single-cell bisulfite sequencing (scBS-seq) technology to characterize partial methylation domains (PMDs) within individual cells of colorectal cancer. The results revealed that over half of the genome was covered by PMDs, and Gain-PMDs, a specific subtype, exhibited a higher coverage of protein-coding genes. Furthermore, the study unveiled substantial epigenetic heterogeneity among different cells within the same tumor and demonstrated that DNA methylation in cells is influenced by the tumor microenvironment [97].

Another study emphasizes the significance of genetic and epigenetic heterogeneity within tumors and its impact on the evolutionary trajectory of cancer. The researchers utilized single-cell bisulfite sequencing analysis (MscRRBS) to investigate this heterogeneity in chronic lymphocytic leukemia (CLL). Their findings revealed that CLL cells exhibit high rates of epigenetic mutations, while showing minimal variation in mutation rates among individual cells. Through comprehensive single-cell analyses, the study elucidated the lineage diversity of CLL cells and their evolutionary patterns following treatment. Notably, the researchers observed specific lineage biases during therapy. By integrating genetic, epigenetic, and transcriptional information at the single-cell level, this study reconstructed the genealogical history of CLL, thereby providing valuable insights into the understanding of tumor development [98].

There are also articles that investigated the DNA methylation profiles of circulating tumor cells (CTCs) using the scBS-seq technique and revealed the subclonal structure, evolutionary history and classification of tissue-specific DNA methylation profiles in CTCs. The results indicate the heterogeneity of DNA methylation in CTCs and reveal the epigenetic regulatory mechanisms in cancer metastasis [99].

Prior epidemiological studies have established a significant association between the consumption of food contaminated with aflatoxin B1 and the incidence of hepatocellular carcinoma. Scientists utilized single-cell RRBS technology to investigate the hepatotoxic mechanism induced by aflatoxin B1 (AFB1) in S phase-arrested L02 cells. The study found that AFB1 caused apoptosis and S phase arrest in L02 cells, reduced mitochondrial membrane potential, increased reactive oxygen species generation, and led to an increase in DNA methylation levels. Through single-cell RRBS analysis, it was revealed that DNA methylation, regulated by the gonadotropin-releasing hormone receptor pathway, Wnt signaling pathway, and TGF-beta signaling pathway, was involved in the hepatotoxic mechanism induced by AFB1 in S phase-arrested L02 cells [1].

Hannah Demond et al. utilized single-cell bisulfite sequencing (scBS-seq) to investigate DNA methylation abnormalities caused by maternal effect mutations in the subcortical maternal complex (SCMC) of human oocytes. These mutations are associated with early embryonic failure, gestational abnormalities, and recurrent pregnancy loss. The researchers observed a genome-wide deficiency of DNA methylation in the oocytes of patients with SCMC mutations compared to normal oocytes. Both the germline differentially methylated regions (gDMRs) of imprinted genes and other sequence features that are normally methylated in oocytes were affected, indicating a lack of selectivity toward imprinted genes. The degree of methylation loss varied across different genomic features. Furthermore, analysis of a preimplantation embryo and molar tissue from the same patient revealed persistent methylation defects at imprinted genes after fertilization, while non-imprinted regions of the genome showed near-normal methylation levels after implantation. These findings emphasize the critical role of the SCMC in de novo methylation in the female germline and provide valuable insights into imprinting defects and potential therapeutic strategies [100].

Nevertheless, single-cell methylation sequencing does have limitations. Firstly, due to the limited amount of DNA within a single cell, single-cell sequencing often faces challenges of low coverage and sparse results. Secondly, compared to traditional bulk cell sequencing, single-cell sequencing may exhibit higher technological noise. For example, the DNA amplification process can introduce biases, resulting in oversampling or undersampling of certain methylation sites and impacting the final analysis results. Additionally, during the single-cell collection process, there may be loss of cellular information, such as spatial relationships between cells, and some cells may not be successfully sequenced due to isolation process-related damage. In conclusion, single-cell methylation sequencing is crucial in uncovering the methylation heterogeneity within individual cells. However, challenges and opportunities for improvement remain in terms of throughput, sequencing depth, and accuracy. As technology continues to advance and improve, we can expect wider applications of single-cell methylation sequencing in life science research.

Table 3 presents the unique advantages and limitations of single-cell DNA methylation in terms of characteristics, throughput, and applicability.

### Histone modification

Within a cell, genomic DNA is not just a string of linear sequences but exhibits a highly complex three-dimensional (3D) spatial structure. This structure is largely dependent on histones, octamers composed of two units each of four core components (H2A, H2B, H3, H4) [101]. These histones possess protruding “tail” structures, which can be regulated via a series of chemical modifications.

Histone modifications represent a key epigenetic mechanism, encompassing, but not limited to, acetylation, methylation, phosphorylation, glycosylation, ubiquitination, and nitrosylation. These modifications are usually catalyzed by specific enzymes, such as histone methyltransferase (HMT) and histone acetyltransferase (HAT). These enzymes add specific chemical groups to the amino acid residues of the histones or remove them, thus altering the state of the chromatin [102].

HMT and HAT can be perceived as “writers,” responsible for adding chemical marks. In turn, histone demethylase (HDM) and histone deacetylase (HDAC) can be seen as “erasers,” tasked with erasing these marks [103]. This series of modifications affects the compactness of the chromatin structure, thereby changing its interaction with DNA, and ultimately leading to the activation or silencing of genes.

**Fig. 6:**
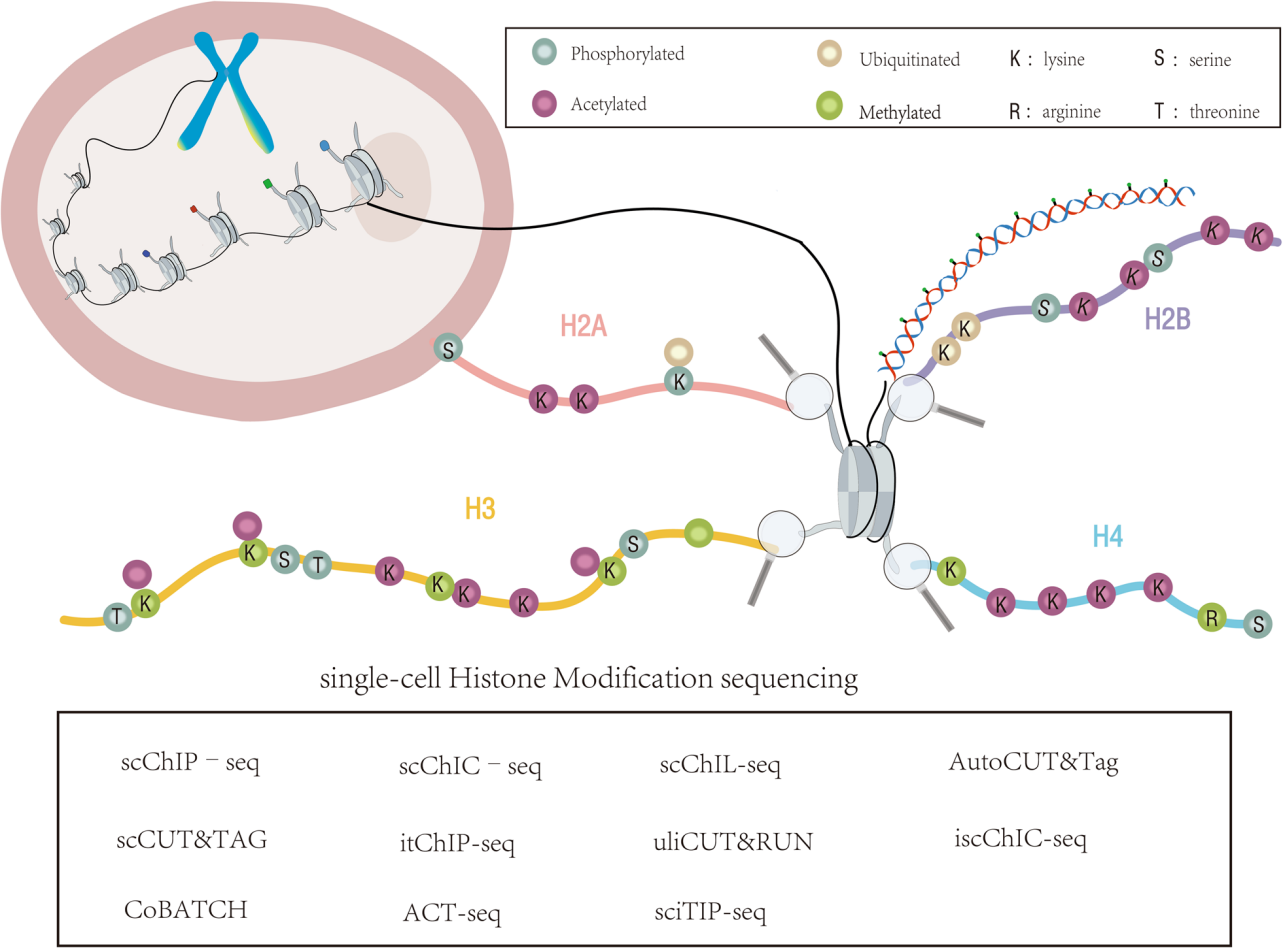
Insights into the dynamic regulation of Histone Modification and methods for single-cell Histone Modification sequencing

The advent of next-generation sequencing (NGS) technologies has been a significant leap forward in understanding gene regulation and epigenetic mechanisms. Early techniques such as chromatin immunoprecipitation (ChIP) microarrays paved the way for more advanced methods, most notably Chromatin Immunoprecipitation Sequencing (ChIP-Seq). Compared to its predecessors, ChIP-Seq offers unparalleled resolution down to the single base-pair level, reduced methodological artifacts, and comprehensive genomic coverage [104]. ChIP-Seq is the gold standard for genome-wide analyses of DNA–protein interactions, histone modifications, and nucleosome positioning. While it offers a wealth of data, traditional or ‘bulk’ ChIP-Seq falls short in assessing the variability in chromatin states across individual cells. This limitation was addressed in 2015, when a landmark paper by David Weitz and Bradley E Bernstein in Nature Biotechnology introduced the concept of single-cell ChIP-Seq108. This innovation permitted the analysis of histone modifications at the single-cell level, thereby unveiling a new dimension of chromatin state heterogeneity. Subsequent to this pioneering work, various methods have emerged to probe chromatin states at single-cell resolution. Techniques such as scCUT&Tag [105], CoBATCH [106], and scChIC-seq [107] have further refined our understanding of chromatin dynamics. Each of these methods summarized in Table 4 and Fig. 6.

**Table 4:** Current methods available for single-cell Histone Modification sequencing

Technique	Technical features	Designed by
scChIP-seq	scChIP-seq is used to study the localization and analysis of protein-chromatin interactions on specific chromatin in a single cell; it features single-cell resolution, chromatin localization, and multi-omics correlation; higher signal-to-noise ratios; technological complexity	[108]
scCUT&Tag	scCUT&Tag is a single-cell chromatin analysis technique for studying chromatin characteristics and transcription factor-chromatin interactions in individual cells; with single-cell resolution, rapid acquisition of chromatin information, and low amplification bias; experimental complexity; data noise;	[105]
CoBATCH	CoBATCH is a sequencing technology based on combined barcoding and targeted chromatin release for single-cell analysis of protein-DNA interactions	[106]
scChIC–seq	scChIC-seq is a method for studying chromatin 3D interactions in individual cells; direct measurement of DNA interactions; combines chromatin immunoprecipitation and chromatin conformation capture techniques to obtain high resolution; technical complexity; data complexity	[107]
itChIP-seq	itChIP-seq is a technique used to study intracellular chromatin characterization; specific antibodies selectively immunoprecipitate target chromatin modifications; preservation of intracellular chromatin organization and spatial information; error and noise depending on the specificity of the antibody and the abundance of chromatin modifications; technical complexity	[109]
ACT-seq	ACT-seq is a high-throughput sequencing technology for analyzing chromatin openness and accessibility; features chromatin accessibility analysis, high-resolution and high-throughput sequencing; feasibility is limited by tissue source and cell type; experimental complexity	[110]
scChIL-seq	scChIL is a method that amplifies genomic sequences closely related to target molecules prior to cell lysis Immunoprecipitation-free epigenomic analysis method	[111]
uliCUT&RUN	uliCUT&RUN is a technology for the study of chromatin and genome-related features; featuring low-input samples, high resolution, and direct sequencing; applied to chromatin modification analysis, transcription factor binding site identification, and cell type identification and phenotyping	[112]
sciTIP-seq	sciTIP-seq is a labeling-based, linear amplification and combinatorial indexing method for mapping histone modifications of individual cells to transcription factor CTCF binding sites	[113]
AutoCUT&Tag	AutoCUT&Tag is a technology for high-throughput, automated chromatin accessibility sequencing; high-throughput, automated, high-resolution; high throughput	[114]
iscChIC-seq	scChIC-seq is a technique for the analysis of chromatin interactions in individual cells; other information (chromatin modifications, transcript determinations) can be obtained simultaneously; there are limitations in signal-to-noise ratios; technological complexity; applications in gene regulation studies, three-dimensional genome structure, and developmental studies	[115]

The significance of single-cell protein modifications lies in revealing the identity and differentiation state of cells, unraveling cellular heterogeneity, studying disease mechanisms, and guiding treatment response and drug discovery. Analyzing protein modification patterns in individual cells helps to understand cellular function and regulation, identify cell-to-cell differences, and provide insights into disease mechanisms for potential therapeutic targets.

A study utilized high-throughput single-cell ChIP-seq technology to investigate chromatin heterogeneity and drug resistance in breast cancer. The researchers employed a microdroplet microfluidic platform to sequence and analyze the chromatin states of thousands of individual cells at single-cell resolution. The study revealed that in untreated drug-resistant tumors, there exists a subset of cells that share chromatin markers with drug-resistant cells, and these cells have lost the chromatin marker H3K27me3 associated with genes promoting drug resistance. This single-cell ChIP-seq technology offers a novel approach to studying the role of chromatin heterogeneity in cancer and other diseases, and aids in uncovering the regulatory mechanisms involved in cellular differentiation and development [116].

There are also study through scCUT&Tag method, combined with scalable nanopore and droplet-based single-cell platforms, was employed to analyze specific chromatin regions in individual cells. The focus was on analyzing the polycomb group (PcG) silencing regions marked by the histone modification H3K27me3 as an orthogonal approach to identify cell states based on chromatin accessibility. Results showed that scCUT&Tag analysis of H3K27me3 could distinguish different cell types in human blood and generate cell type-specific PcG landscapes in heterogeneous tissues. Furthermore, the study utilized scCUT&Tag to analyze H3K27me3 in brain tumor patients before and after treatment, identifying cell types in the tumor microenvironment and revealing heterogeneity in PcG activity between primary samples and post-treatment [117].

Another study utilized automated CUT&Tag chromatin profiling to investigate the impact of KMT2A oncofusion proteins in leukemias. By mapping fusion-specific targets across the genome, the researchers identified common and tumor-subtype-specific sites of aberrant chromatin regulation. They found that certain binding sites for KMT2A oncofusion proteins exhibited cell-to-cell heterogeneity and were associated with lineage plasticity. Additionally, they discovered that abnormal enrichment of H3K4me3 in gene bodies could be targeted by Menin inhibitors. The integration of automated and single-cell CUT&Tag techniques enabled the identification of epigenomic heterogeneity within patient samples and the prediction of therapeutic sensitivity [114].

Over the years, we have gradually come to recognize the complexity and importance of histone modifications and chromatin states in gene regulation. The emergence of single-cell technologies is a significant breakthrough, allowing us to explore previously uncharted layers of epigenetic regulation. New technologies are expected to emerge that integrate the advantages of existing methods, providing higher resolution and throughput, and possibly reducing costs as well. Furthermore, the continued development of computational analysis and machine learning will help in parsing increasingly complex datasets. In short, the field of single-cell histone modification is still evolving and holds the promise of providing us with a deeper understanding of gene regulation. It may also inspire our insights into cellular processes and disease mechanisms.

Table 4 presents the unique advantages and limitations of Histone Modification sequencing in terms of characteristics, throughput, and applicability.

### Nucleosome localization

Nucleosome positioning refers to the precise localization of nucleosomes, which are structural units composed of an octamer of histone proteins and the wrapped DNA, on the genome [109]. In eukaryotic chromosomes, the binding of DNA to histones is not static, and the accurate determination of nucleosome positions on the genome, known as nucleosome positioning, is crucial for maintaining genome structure and function.

The regulation of nucleosome positioning is closely associated with the spatial organization of chromatin, DNA replication, transcription, and gene expression regulation [118]. Nucleosome positioning can influence the chromatin state and accessibility, thereby impacting the transcriptional activity of genes. The positions of nucleosomes on the genome can affect the binding of transcription factors and the accessibility of promoters, determining the transcriptional levels and patterns of genes.

The study of nucleosome positioning can be conducted using various experimental techniques and computational methods. Among them, micrococcal nuclease sequencing (MNase-seq) is a widely used approach that involves the digestion of chromatin with micrococcal nuclease to degrade nucleosome structures, releasing the core DNA of nucleosomes, which can then be sequenced using high-throughput sequencing technology [56]. By analyzing the sequencing data, the positions and positioning patterns of nucleosomes can be determined.

Recent studies have demonstrated that even within a homogeneous population of cells, different cells exhibit significant heterogeneity in chromatin states [119, 120], which may be related to heterogeneity in chromatin accessibility. scMNase-seq is a high-throughput sequencing technique used for studying nucleosome positioning at the single-cell level. It can uncover the heterogeneity of nucleosomes between cells and investigate cell-specific chromatin states and gene regulatory mechanisms [56].

In one research, through single-cell MNase-seq analysis, researchers identified distinct accessible chromatin regions in all lymphoid progenitor cells (ALP), early ILC progenitors (EILP), and ILC progenitors (ILCP). Within EILP, different subpopulations were identified, indicating their potential to differentiate into either dendritic cell lineage or ILC lineage based on epigenetic profiles. The researchers found that the transcription factors TCF-1 and GATA3 co-bound with lineage defining sites (LDS-Is) associated with ILC, while PU.1 binding was enriched in LDS (LDS-As) associated with alternative dendritic cell fate. TCF-1 and GATA3 were found to be essential for the epigenetic priming of LDS at the EILP stage. These findings suggest that the fate of multipotent progenitors during the differentiation into ILC and hematopoietic stem cells is pre-defined by their epigenetic state. The presence of distinct subpopulations within multipotent progenitors and their regulation by key transcription factors highlight the heterogeneity of cells that contribute to lineage specification. The application of single-cell MNase-seq technology enables a deeper understanding of the epigenetic changes during cellular differentiation, shedding light on the molecular mechanisms and role of transcription factors in determining cell fate [121].

## Genomics

With the advancement of technology, researchers are able to study cells in a more detailed manner through methods such as single-cell genomics sequencing, rather than relying on bulk tissue samples alone. This provides us with a unique perspective that allows us to gain a deeper understanding of the underlying mechanisms of complex diseases, particularly cancer. Cancer can be understood as a genomic disorder in which the accumulation of somatic mutations and epigenetic modifications plays a crucial role in its development. Somatic mutations include various types of genomic alterations introduced into the genome, such as single nucleotide variants (SNVs), structural variations, gene splicing variants, and copy number variations [122]. These genomic alterations contribute to the occurrence of cancer and other diseases. Considering the frequencies of these changes in the human genome, it becomes particularly important to comprehensively understand the gene expression patterns of individual cells.

Traditional genomics sequencing involves sequencing DNA or RNA from populations or tissues. However, this method typically results in the average sequencing of the genome from the population and loses the genetic information of individual cells, as well as cellular heterogeneity, the ability to detect rare variations, distinguish between cell types, and lack of cellular functional information [123].

To overcome these limitations, the field of single-cell genomics (SCG) has emerged as an important technology within the genomics field, aiming to perform high-throughput sequencing of individual cells' genomes to reveal their genetic characteristics and structures, providing a new approach to understanding tumor development and disease progression [124]. Through single-cell genomics sequencing, researchers can obtain genetic information about individual cells, including gene mutations, copy number variations, chromosomal structures, and rearrangements. Additionally, it can reveal important features such as genomic-level cellular heterogeneity, cell type differentiation, and development.

Currently, the most common application of SCG is the analysis of copy number variations (CNVs). For example, in a study on hepatocellular carcinoma (HCC), single-cell DNA sequencing (scDNA-seq) revealed that the accumulation of CNVs follows a biphasic copy number evolution model, confirming for the first time at the single-cell level that multiploid hepatocellular carcinoma originates from diploid cells [125]. Another study examining colorectal cancer patients found that fibroblasts with somatic copy number alterations (SCNAs) in tumor tissues were significantly more abundant than in adjacent normal tissues, suggesting potential functional consequences or effects of these chromosomal-level SCNAs in tumor development. Fibroblasts with SCNA may interact with cancer cells, contributing synergistically to tumor development [126].

Mutation detection is another significant application of single-cell genomics (SCG), which holds great potential but can be costly. To enhance cost-effectiveness, a strategic approach involves initially conducting bulk sequencing, followed by targeted sequencing with increased depth focusing on specific loci of interest for mutation analysis. This sequential approach allows for a broader survey of genomic information through bulk sequencing, followed by a more in-depth analysis of specific regions of interest, optimizing both resource utilization and mutation detection efficiency.

This article employs single-cell mutational profiling to investigate myeloid malignancies, particularly acute myeloid leukemia (AML). The study reveals that AML is primarily driven by a small number of dominant clones, often carrying co-occurring mutations in epigenetic regulatory genes. Conversely, mutations in signaling genes tend to occur in distinct subclones, contributing to increased clonal diversity. Through single-cell analysis, interactions between mutations are unveiled, along with the relationship between immunophenotype and somatic genotype/clonal architecture. This research provides valuable insights into the pathogenesis of myeloid malignancies and the evolution of disease progression.

With the continuous advancement of sequencing technologies, single-cell whole-genome sequencing (scWGS) has become possible. scWGS is a technique for analyzing the DNA of individual cells, enabling the sequencing of their complete genomes at the single-cell resolution [127]. It involves amplifying the DNA of single cells through methods such as multiple displacement amplification (MDA) or polymerase chain reaction (PCR), and the amplified DNA is then subjected to high-throughput sequencing technologies such as Illumina sequencing. The sequencing data obtained through scWGS can provide valuable information about the genomic features of individual cells within heterogeneous cell populations. It can help identify genetic variations within single cells, including single nucleotide variants (SNVs), copy number variations (CNVs), and structural variations (SVs).

For example, a study utilized scWGS to analyze recurrent liver metastatic lesions in patients with metastatic colorectal cancer. The study found that treatment resulted in a severe reduction in the number of tumor cells in the liver metastatic lesions, but previously differentiated cell lineages remained alive and potentially survived through migration to different sites within the liver. These cell lineages underwent slow evolution under adjuvant drug treatment for 2 years and then rapidly diversified within a short period of time. The study also identified several non-silent mutations specific to these cell lineages and speculated that chemotherapy contributed significantly to the overall genomic mutational burden. Overall, the study revealed that subclones of metastatic colorectal cancer could undergo local migration and escape surgical resection, continue to evolve under chemotherapy, and exhibit explosive re-expansion [128].

Another study used scWGS to analyze cells from a Chinese female patient with cervical cancer before and after radiotherapy. They discovered a deleterious mutation in the NFKB12 gene that increased in frequency after radiotherapy. Functional analysis revealed that NFKB430 acts as a tumor suppressor, and the mutation in NFKB1 weakened its tumor suppressive function. NFKB1 enhanced radiation sensitivity, and its mutation reduced this effect. The study suggests that NFKB1 could be a potential molecular target for future cervical cancer radiotherapy [129].

However, scWGS has certain limitations when it comes to detecting copy number variations (CNVs), such as lower accuracy and insufficient amplification fidelity. To overcome these limitations, a series of new techniques, including MALBAC, eMDA, LIANTI, SISSOR, and META-CS, have emerged to improve the accuracy of CNV detection and reduce false-positive rates. Table 5 provides an overview of single-cell genomics sequencing methods.

**Table 5:** Current methods available for single-cell genomic sequencing

Technique	Technical features	Designed by
DOP-PCR	DOP-PCR is a polymerase chain reaction (PCR) method that uses specific oligonucleotide primers and DNA polymerase to amplify specific DNA sequences; high amplification efficiency; applicable to DNA samples from a variety of sources, including genomic DNA and cDNA; may be over-amplified or selectively amplified; may cause non-specific amplification; restrictive selection	[130]
MDA	MDA is a technique for whole-genome amplification in low-complexity DNA samples; has whole-genome amplification, allele retention, and no need for specific primers; has limitations of amplification bias, localized error rates, and sample contamination; and costs low	[131]
LIANTI	LIANTI is an improved single-cell whole-genome amplification (WGA) method that accurately detects copy-number variations (CNVs) at a microscale resolution; It enables the observation of DNA replication origin firing patterns and addresses the origin of cytosine-to-thymine mutations in single-cell genomics; advancements in CNV detection, amplification fidelity, and the study of DNA replication and mutation profiles in single cells	[132]
META-CS	META-CS is a single-cell whole-genome amplification method that utilizes the complementarity of double-stranded DNA to accurately identify single-nucleotide variations (SNVs); the ability to amplify diploid and haploid cells, high success rate of single-cell amplification, simplified experimental procedure, and reduced sequencing cost; false positive mutations and inconsistent amplification efficiency	[133]
MALBAC	MALBAC is a single-cell whole genome amplification technique for amplifying DNA from a single cell; reduced amplification randomness and bias, increased homogeneity of amplification products; low error rate; limitations of reduced amplification quality, loss of complexity, and amplification bias	[134]
eWGA	eWGA is an enhanced whole-genome amplification technique used to amplify DNA samples starting from very low quantities to obtain sufficient amounts of DNA for subsequent analysis; characterized by high amplification efficiency, low amplification bias, homogeneity, and accuracy; limitations of preferential amplification, localized error rates, and co-amplification contamination; and relative economy	[135]
SISTOR	SISSOR is a method used for precise sequencing of single-cell genomes and haplotype analysis; based on a microfluidic processor to separate and amplify the DNA strands in individual cells; This enables independent sequencing of complementary strands and assembly of long haplotypes; low error rates and can generate DNA fragments that can be assembled into haplotype contigs	[136]
ScWGS	scWGS is a method that enables deep sequencing of the genomic DNA of individual cells; based on microfluidic technology and DNA amplification techniques; relatively lower throughput; capture mutations present in different cells, study rare events, not require a large amount of starting material; amplification bias, complex data analysis; cost high	[137]
scWES	scWESis a targeted sequencing method that allows for the sequencing of the exome, including the coding regions of the genome, within individual cells; provides a comprehensive view of the genetic variations present in the coding regions of single cells, including single nucleotide variants (SNVs), small insertions or deletions (indels), and structural variations; relatively lower throughput; amplification biases and technical noise; relatively expensive; studying cellular heterogeneity	[138]
scDNA-seq	scDNA-seq allows for the whole-genome sequencing of individual cells, providing a comprehensive view of genomic variations, including mutations, copy number variations, and structural variations; relatively low throughput; detecting low-frequency mutations and copy number variations; Limitations include sequencing depth constraints, potential amplification biases, and technical noise; relatively expensive; data complex; reveals information about genetic heterogeneity between cells and cell evolution	[139]

Table 5 presents the unique advantages and limitations of single-cell genomic sequencing in terms of characteristics, throughput, and applicability.

## Multi-omics

Recent advances in single-cell genomics technology have allowed us to analyze the epigenome, genome, and transcriptome of individual cells with single-cell resolution, greatly enhancing our ability to study tumor heterogeneity. Tumors can be viewed as ecosystems with complex interactions between different phenotypes [140]. However, analyzing cells at the individual level may only provide a partial picture of the intricate regulatory networks, limiting our understanding of the mechanisms driving cancer initiation and progression. Currently, advanced analysis techniques and experimental methods are being developed to effectively capture information from multiple epigenomic layers within individual cells. By integrating epigenomics with other omics technologies, we can gain a more comprehensive understanding of these intricate interactions, thus unraveling the nature of tumor heterogeneity. In cancer research, the epigenome, transcriptome, and genome exhibit significant heterogeneity. The integration of single-cell epigenomics with multi-omics analysis holds promise in better elucidating the mechanisms driving cancer initiation and development, as well as the regulatory heterogeneity [141] (Table 6).

**Table 6:** Current methods available for single-cell multi-omics sequencing

Technique	Technical features	Designed by
Paired-Tag	Paired-Tag is a high-throughput genomic sequencing technology that enables simultaneous sequencing of target DNA and its adjacent regions; high throughput; relatively low cost; comprehensive sequencing information; applicability in gene structure analysis, genomic variation analysis, and chromatin conformation studies; requires rigorous primer design; complex data analysis	[155]
Paired-seq	Paired-seq is a high-throughput sequencing technique that integrates scRNA-seq and scDNA-seq to enable simultaneous analysis of the transcriptome and genome of cells; high throughput; relatively higher costs; requires complex primer design and data analysis;	[1]
scDAM&T-seq	scDAM&T-seq is a high-throughput sequencing technique that combines single-cell DNA adenine methylome and transcriptome sequencing; simultaneous analysis of the DNA adenine methylation and gene expression at the single-cell level; high throughput; relatively higher costs;	[156]
sci-CAR-seq	sci-CAR-seq simultaneously analyzes the transcriptome and chromatin conformation information of individual cells; simultaneous view of gene expression and chromatin three-dimensional structure at the single-cell level; high throughput; relatively higher costs	[144]
SHARE-seq	SHARE-seq allows for the simultaneous analysis of the transcriptome and chromatin interactions; high throughput; relatively higher costs	[145]
SNARE-seq	SNARE-seq can simultaneously obtain transcriptome and chromatin accessibility data from the same individual cell; it is suitable for tissues that are difficult to separate into single-cell suspensions; Microfluidic-based Single-Cell Isolation and Barcoding Technology; medium to high throughput	[146]
ASTAR-seq	ASTAR-seq is a new single-cell sequencing technology that simultaneously measures the transcriptome and chromatin accessibility of cells; multiple cells are processed at the same time in a single experiment, which improves experimental throughput; incompatible with automated platforms; may result in gene loss	[147]
scCAT-seq	scCAT-seq, a technique for simultaneous determination of chromatin accessibility and transcriptome within the same single cell; accurate construction of regulation between cis-regulatory elements and target genes	[143]
scMT-seq	scMT-seq is a composite sequencing technology that allows for simultaneous analysis of the genome, transcriptome, and methylome of individual cells; lower throughput, higher cost, and complex operation; reveals the interactions between different biological molecular levels within single cells	[151]
scmCT-seq	Methylome + transcriptome	[152]
scM&T-seq	scM&T-seq is a technology that enables the simultaneous acquisition of DNA methylation and transcriptome information from individual cells; relatively costly and technically complex	[153]
scGEM	scGEM is a single-cell genome and epigenome sequencing technology that allows for the simultaneous analysis of the genome and epigenome of individual cells; relatively costly and technically complex; offers high throughput and reveals genetic and epigenetic heterogeneity among cells	[154]
Pi-ATAC	Pi-ATAC combines CRISPR-Cas9 gene editing technology with ATAC-seq (Assay for Transposase-Accessible Chromatin with high-throughput sequencing) to enable site-specific genome editing while simultaneously measuring the impact on genome accessibility; high throughput capacity; higher cost; technically complex	[162]
ASAP-seq	ASAP-seq is a high-throughput sequencing technique that combines single-cell DNA adenine methylome and transcriptome sequencing; simultaneous analysis of the DNA adenine methylation and gene expression at the single-cell level; high throughput; relatively higher costs	[163]
scNMT-seq	scNMT-seq allows concurrent profiling of nucleosome structure, DNA methylation, and transcriptome information from individual cells, providing comprehensive analysis across different cellular levels; high throughput capabilities; higher costs; technical complexity	[157]
scNOMeRe-seq	scNOMeRe-seq is a sequencing technique that allows for the simultaneous analysis of nucleosome occupancy and DNA methylation information from individual cells; high throughput; high costs and complex data analysis; operationally and analytically complex	[158]
scChaRM-seq	scChaRM-seq is a sequencing technique that allows for simultaneous profiling of chromatin accessibility and DNA methylation information from individual cells; high throughput; high costs; technical complexity	[159]
snmCAT-seq	snmCAT-seq is a sequencing technique that allows for the simultaneous measurement of multiple omics information, including gene expression, chromatin accessibility, and DNA methylation, from individual cell nuclei; high throughput; utilizes a combination of multiple molecular markers, such as RNA, DNA methylation, and chromatin accessibility tags, to capture the multi-dimensional; research cell typing, cell type classification, and functional analysis; complex experimental procedures	[160]
Smart-RRBS	Smart-RRBS can simultaneously obtain transcriptome information and copy number alteration (CNA) information; technical complexity; high throughput	[149, 150]
scNOME-seq	scNOME-seq is a single-cell technology used to determine the assembly of nucleosomes and DNA methylation patterns within cells; detection of subtle variations in DNA methylation patterns; relatively low throughput; limited by sequencing depth restrictions	[164]
scMethyl-HiC	scMethyl-HiC is a technology that combines single-cell DNA methylation sequencing with chromosome conformation capture (Hi-C) to simultaneously detect DNA methylation and chromosomal spatial structure information in individual cells; insights into the heterogeneity of DNA methylation and spatial structure between cells; relatively low throughput; higher costs; complex experimental workflow and data analysis requirements; high sequencing depth requirements and a complex experimental process	[164]
sn-m3C-seq	sn-m3C-seq is a single-nucleus multi-omics methylcytosine and chromatin conformation sequencing technology that enables simultaneous measurement of DNA methylation and chromatin 3D structure information in individual cells; reveals the heterogeneity of DNA methylation and chromatin structure among cells, providing insights into cellular variability; relatively low throughput; higher costs; complex experimental workflow and data analysis	[165]
scCOOL-seq	scCOOL-seq is a single-cell chromatin conformation and open chromatin sequencing technology used for simultaneously measuring the chromatin 3D structure and open chromatin regions in individual cells; low throughput; higher costs; exploration of the relationship between chromatin structure and gene regulation in individual cells; complex experimental workflow and data analysis	[166]
iscCOOL-seq	iscCOOL-seq is an improved single-cell COOL-seq method based on the TAILS strategy. iscCOOL-seq can simultaneously analyze chromatin accessibility, DNA methylation, and gene expression; sequencing depth and coverage are limited; and sample processing and experimental manipulation are demanding	[167]

In recent years, multi-omics technologies have emerged as crucial tools for deciphering the complexity of biological systems, particularly at the single-cell level. Independently, single-cell transcriptomics and single-cell epigenomics provide rich information about gene expression and epigenetic regulation. However, integrating these two single-cell-level holistic analytical methods has the potential to reveal deeper insights. This integration is especially critical in the context of tumor biology. Individually, these technologies can describe the transcriptional states, expression profiles, and epigenetic features of individual cells within tumor tissues. However, by jointly analyzing these two types of data, we can not only identify epigenetic patterns within specific cell populations but also unravel how these patterns directly influence or are influenced by transcriptional activity. Specifically, such multi-omics analysis enables us to explore in detail how epigenetic regulation affects the maintenance and transition of cell identity. Furthermore, it helps us understand how these epigenetic mechanisms manipulate cellular fate, driving cells into specific lineage trajectories and generating varying levels of heterogeneity within tumor tissues [142].

For example, scCAT-seq, which stands for single-cell chromatin accessibility and transcriptomics sequencing, is an advanced multi-omics technique that enables simultaneous analysis of chromatin accessibility and gene expression within individual cells [143]. It combines the strengths of single-cell chromatin accessibility analysis such as ATAC-seq or DNase-seq, with single-cell RNA sequencing (scRNA-seq), providing a comprehensive view of the epigenetic and transcriptional landscape within single cells. By integrating chromatin accessibility and transcriptomic data obtained from scCAT-seq, researchers can study the relationship between chromatin structure and gene expression at single-cell resolution. This integrative analysis allows for the identification of regulatory elements involved in specific transcriptional programs and characterization of cellular heterogeneity based on epigenetic and transcriptional states. The scCAT-seq technique successfully acquired precise chromatin accessibility (CA) and gene expression (GE) information in lung, cervical, and colorectal cancers [143]. Other tools for joint transcriptome chromatin accessibility analysis include sci-CAR-seq [144], SHARE-seq [145], SNARE-seq [146], ASTAR-seq [147], and Joint scATAC-Seq/scRNA-seq [148].

Smart-RRBS is an advanced single-cell multi-omics technique that provides single-cell-level information on genomic DNA methylation and transcriptome. This technology is of great significance in exploring cellular heterogeneity, developmental processes, and epigenetic regulation in disease mechanisms [149, 150]. By integrating genomic DNA methylation and gene expression data, Smart-RRBS enables in-depth analysis of the functionality and regulatory patterns of gene regulatory networks within cells.

One notable study utilized this technique to examine chronic lymphocytic leukemia (CLL) and glioblastoma cells, uncovering the crucial role of intratumoral epigenetic heterogeneity in cancer progression. For instance, Gaiti et al. [150] employed Smart-RRBS to map CLL lineage history and predict its evolution after therapy by leveraging epigenetic information. Another study by Chaligne et al. focused on glioma cells, revealing the heritability of epigenetic patterns within this population. They discovered distinct variations in cell plasticity states between IDH-mutant and IDH-wild-type glioblastoma, demonstrating the significance of epigenetic traits in differentiating glioblastoma subtypes [1]. Methods for simultaneous analysis of DNA methylome and transcriptome from the same cell at single-cell resolution also include scMT-seq [151], scmCT-seq [152], scM&T-seq [153], and scGEM [154]. Single-cell transcriptomes can also be combined with histone modifications and protein-DNA interactions, such as Paired-Tag [155], scDAM&T-seq [156].

The regulation of gene expression involves a dynamic interplay in vivo among various epigenomic layers, including DNA methylation, chromatin accessibility, and others, rather than functioning independently of one another. cNMT-seq [157], scNOMe-seq [158], scChaRM-seq [159], and snmCAT-seq [160] can combine single-cell transcriptomics and multiple epigenomic layers to analyze the relationship between multiple epigenetic features and gene expression at single-cell resolution and play a crucial role in further understanding the epigenetic-dependent associations on transcription and cellular states. For instance, Bian et al. used scTrio-seq to study colorectal cancer tumors and metastases from 10 patients. They found that DNA methylation levels were consistent within genetic lineages but varied significantly among clones. The technique provided insights into tumor evolution and the relationship between DNA methylation and genetic lineages. Overall, scTrio-seq allowed for a comprehensive analysis of mutations, transcriptome, and methylome in individual cells, shedding light on tumor heterogeneity and metastasis [161].

There are many other tools that can combine single-cell proteomics with single-cell epigenomics such as Pi-ATAC [162] and ASAP-seq [163]. Pi-ATAC can identify both epigenomic and proteomic heterogeneity in a single cell. A study by quantifying the protein levels of Pi-ATAC on TFs NF-kB and HIF1α in mouse mammary tumors and measuring the DNA occupancy of both found that the primary role of HIF1α protein in the tumor microenvironment of tumor hypoxia is through shaping the regulatory groups in parenchymal tumor cells and infiltrating immune cell subpopulations [162].

Some tools can explore the epigenome of several layers of a single cell simultaneously to achieve a more comprehensive epigenomic analysis, such as scNOME-seq (chromatin accessibility + DNA methylation status) [164], scMethyl-HiC [164] and sn-m3C-seq (chromatin 3D structure + methylome) [165].

Other multi-omics analysis solutions include scCOOL-seq [166] and iscCOOL-seq [167] that allow simultaneous analysis of chromatin state/nucleosome localization, DNA methylation, copy number variation and chromosome ploidy in individual cells, enabling the combination of different epigenomic sequencing layers in a single cell at the same time. Although integrated multi-omics technologies such as iscCOOL-seq in single-cell analysis have tremendous potential in exploring the interplay between multiple layers of the epigenome and the genome within individual cells, their application in tumor research is still not widely adopted. This relatively new technology faces several challenges and limitations. Firstly, the maturity of these techniques is relatively low, requiring more laboratories to become familiar with and master the experimental and data analysis workflows. Secondly, the experimental procedures of these techniques are complex and require optimization of experimental conditions and coordination of multiple steps. Additionally, the complexity of data interpretation and analysis poses a challenge, necessitating the development of more reliable multi-omics data analysis methods and resources. Lastly, sample requirements and feasibility are also limiting factors for the application of these technologies, particularly in obtaining a sufficient quantity and quality of single-cell samples from tumors. Despite these challenges, we can anticipate further research efforts to overcome the technical challenges, improve data interpretation and analysis, and expand feasibility, thereby promoting the widespread application of technologies like iscCOOL-seq in tumor research to uncover mechanisms of tumor development and facilitate precision therapeutics.

Table 6 presents the unique advantages and limitations of multi-omics sequencing in terms of characteristics, throughput, and applicability.

## Concluding remarks and future perspectives

Nowadays, cancer medicine has entered the era of precision medicine, and single-cell sequencing technologies based on next-generation sequencing are rapidly advancing. Traditional bulk sequencing methods often provide analysis based on the average values of multiple cells, which cannot provide a high-resolution view of the cellular composition in the tumor ecosystem. Compared to bulk sequencing methods that provide averaged data, single-cell sequencing has significant advantages. It allows analysis of the transcriptome, genome, and epigenome of individual tumor cells, revealing the heterogeneity within the tumor.

The interplay of various "omics" within a cell follows a complex regulatory mechanism, starting from the genome and epigenome, extending to the transcriptome, proteome, and back again [22]. This intricate process highlights the crucial role of epigenetic regulation in the gene regulatory network. Cancer can be viewed retrospectively as an epigenetic disease, wherein the identity and function of different cell types within various tumor cells are determined by the epigenetic landscape of those cells. Consequently, non-genetic factors significantly contribute to tumor progression [141]. The study of epigenetics has greatly enhanced our understanding of tumor heterogeneity. In recent years, numerous cutting-edge techniques have been developed to analyze epigenetic modifications at the single-cell resolution level. These techniques encompass the examination of chromatin accessibility, DNA/RNA methylation, histone modifications, and nucleosome localization. They provide invaluable research methods for investigating the intricate relationship between tumor epigenetic modifications and tumor heterogeneity.

To comprehensively understand the complex epigenetic regulatory mechanisms driving tumor cell development, progression, and drug resistance within the tumor microenvironment, it is often insufficient to rely solely on single-cell epigenomic analysis. Rather, the integration of multiple omics data at a single-cell resolution is necessary. This entails considering genomic, epigenomic, transcriptomic information.

By employing single-cell multi-omics techniques and analyses, it becomes possible to simultaneously capture multiple layers of epigenetic information together with other omics data. For instance, a commonly used approach involves combining single-cell epigenomics and transcriptomics, allowing researchers to investigate the relationship between the epigenome and gene expression at the genomic level in individual cells [168]. Furthermore, multi-omics studies that incorporate DNA methylation patterns, open chromatin regions, nucleosome positioning, DNA–protein interactions, genomic alterations (mutations and copy number variations), as well as mRNA or protein abundance at the single-cell level, have been employed in tumor heterogeneity research. These comprehensive analyses enable a more in-depth understanding of the epigenome and highlight the significance of epigenetic heterogeneity within tumors in cancer progression [168].

However, the application of single-cell multi-omics analysis to investigate tumor heterogeneity is still in its early stages and faces technical and computational challenges. These challenges include limitations in obtaining valid information, which can result in missing values, systematic noise, and issues with sample coverage. These factors can impact the final analysis of the data, thereby affecting our understanding of tumor heterogeneity.

For example, traditional bisulfite-based methods used to measure single-cell DNA methylation can introduce DNA damage and compromise the accuracy of DNA methylome sequencing. To overcome this limitation, the application of third-generation/real-time single molecule sequencing (TGS) can be employed [169]. TGS has the advantage of directly detecting epigenetic modifications, including DNA 5 mC and 6 mA, without the need for bisulfite conversion or PCR amplification. Additionally, recent advancements in nanopore technology, such as SMAC-seq, Fiber-seq, and nanoNOMe, have enabled long-range detection of single-molecule chromatin states by combining nanopore sequencing with m6A methyltransferase or M. CviPI GpC methyltransferase enzyme accessibility [170–172]. These innovations reduce DNA damage and improve the accuracy of DNA methylome sequencing, providing researchers with a more precise tool for studying epigenetic modifications.

The integration of spatial omics with single-cell omics such as (spatial proteomics, spatial transcriptomics, spatial epigenomics) has improved our understanding of the evolution of cancer in temporal and spatial dimensions. However, the study of tumors by spatial omics is still in its infancy, and the next step is to extend spatial mono-omic to spatial multi-omics by converting spatial information into DNA barcodes, or imaging-based methods to preserve spatial information of each cell [23]. The spatial distribution of epigenome and transcriptome is a key determinant of the cellular characteristics, and the extension of spatial transcriptomic approaches to epigenomic analysis is a critical step forward in our efforts to unravel the epigenetic drivers underlying tumor heterogeneity.

A 3D tumor-like organ model with certain spatial structure constructed by culturing tumor stem cells in vitro has the ability to stably preserve the epigenomic, proteomic, transcriptomic, morphological and pharmacological features of the parent tumor [173]. Although a tumor-like organ is not a genuine human tumor, it has received widespread attention for its ability to simulate real organs structurally and functionally, which closely reflects the pathophysiological characteristics of tumorigenesis and metastasis in humans. With the advent of the era of spatial multi-omics technology, biomarkers based on peripheral blood and bulk tissue biopsies will not be able to meet the demand of transferring laboratory results to medical and clinical research. Tumor-like organs can mimic the real environment of tumors in human body, with the advantages of closer physiological cell composition and behavior, more stable genome, more suitable for biotransfection and high-throughput screening, providing a fast and effective way for tissue spatial biomarker discovery and drug screening. It provides a rapid and effective platform for tissue spatial biomarker discovery and drug screening and offers a validation model for us to explore the key epigenetic mechanisms behind the prognosis and therapeutic resistance of tumor patients.

Although single-cell sequencing has improved our understanding of the relationship between epigenetics and tumor heterogeneity, we have to face the following obstacles. Firstly, due to the large amount of information in the epigenome (tens of fold larger than the transcriptome) which is distributed throughout the genome, the coverage of the epigenome of a single cell by current methods is still relatively thin, and research errors arise when distinguishing technical noise from intercellular differences, and novel biochemistry or strategies may be needed to overcome this limitation in the future. In addition, multi-omics analysis of a single cell at a time, although providing a comprehensive molecular profile, generates very complex data that needs to be analyzed both to match information between omics and to reduce errors and biases generated in data matching, thus requiring simplified and targeted analysis of the data of interest. Finally, large-scale single-cell analysis of complex tumor samples can be expensive due to the low throughput and high single-cell cost of single-cell sequencing technologies. These characteristics determine that single-cell technology still has a long way to go before it can be widely used in clinical cancer research, but it is certain that the future of single-cell epigenetics in clinical applications is bright, and perhaps in the near future, further understanding of single-cell epigenetics will undoubtedly improve our knowledge of tumor cell heterogeneity and the prognosis and drug resistance mechanisms of tumor patients, providing patients with more individualized clinical applications.

## Data Availability

Not applicable; all information in this review can be found in the reference list.

## Abbreviations

TME: Tumor microenvironment
CTC: Circulating tumor cell
CSC: Cancer stem cell
SNV: Single-nucleotide variation
CNV: Copy number variation
Sv: Structural variation
HCC: Hepatocellular carcinoma
M1: Classically activated macrophages
M2: Alternatively activated macrophages
